# Supplementary material for: The monsoon-associated equine South African pointy mosquito ‘Aedes caballus’; the first comprehensive record from southeastern Iran with a description of ecological, morphological, and molecular aspects
Source: PLoS One. 2024 May 23;19(5):e0298412. doi: 10.1371/journal.pone.0298412 (PMC11115297; doi:10.1371/journal.pone.0298412)
Supplement: S1 Fig — COI barcode region with 0.89% diversity, including 66.7% transitions and 33.3% transversions. (PDF) [file pone.0298412.s001.pdf]

```
CLUSTAL O(1.2.4) multiple sequence alignment


P4      ------------AAAGATATTGGTACTTTATATTTTATTTTCGGAGTTTGATCAGGAATA   48
P3      ------------AAAGATATTGGTACTTTATATTTTATTTTCGGAGTTTGATCAGGAATA   48
KC1     ---------------GATATTGGTACTTTATATTTTATTTTCGGAGTTTGATCAGGAATA   45
V2      CAACCAAAACATAAAGATATTGGTACTTTATATTTTATTTTCGGAGTTTGATCAGGAATA   60
V3      ------------AAAGATATTGGTACTTTATATTTTATTTTCGGAGTTTGATCAGGAATA   48
N5      --------------AGATATTGGTACTTTATATTTTATTTTCGGAGTTTGATCAGGAATA   46
KC5     ---------CATAAAGATATTGGTACTTTATATTTTATTTTCGGAGTTTGATCAGGAATA   51
                       *********************************************

P4      GTTGGTACATCATTAAGAATTTTAATTCGTGCTGAATTAAGTCAACCAGGAATATTTATT   108
P3      GTTGGTACATCATTAAGAATTTTAATTCGTGCTGAATTAAGTCAACCAGGAATATTTATT   108
KC1     GTTGGTACATCATTAAGAATTTTAATTCGTGCTGAATTAAGTCAACCAGGAATATTTATT   105
V2      GTTGGTACATCATTAAGAATTTTAATTCGTGCTGAATTAAGTCAACCAGGAATATTTATT   120
V3      GTTGGTACATCATTAAGAATTTTAATTCGTGCTGAATTAAGTCAACCAGGAATATTTATT   108
N5      GTTGGTACATCATTAAGAATTTTAATTCGTGCTGAATTAAGTCAACCAGGAATATTTATT   106
KC5     GTTGGTACATCATTAAGAATTTTAATTCGTGCTGAATTAAGTCAACCAGGAATATTTATT   111
        ************************************************************

P4      GGAAATGACCAAATTTATAATGTAATTGTTACAGCTCATGCTTTTATTATAATTTTCTTT   168
P3      GGAAATGACCAAATTTATAATGTAATTGTTACAGCTCATGCTTTTATTATAATTTTCTTT   168
KC1     GGAAATGACCAAATTTATAATGTAATTGTTACAGCTCATGCTTTTATTATAATTTTCTTT   165
V2      GGAAATGACCAAATTTATAATGTAATTGTTACAGCTCATGCTTTTATTATAATTTTCTTT   180
V3      GGAAATGACCAAATTTATAATGTAATTGTTACAGCTCATGCTTTTATTATAATTTTCTTT   168
N5      GGAAATGACCAAATTTATAATGTAATTGTTACAGCTCATGCTTTTATTATAATTTTCTTT   166
KC5     GGAAATGACCAAATTTATAATGTAATTGTTACAGCTCATGCTTTTATTATAATTTTTTTT   171
        ******************************************************** ***

P4      ATAGTAATACCTATTATAATTGGAGGATTTGGAAATTGATTAGTTCCTCTAATATTAGGA   228
P3      ATAGTAATACCTATTATAATTGGAGGATTTGGAAATTGATTAGTTCCTCTAATATTAGGA   228
KC1     ATAGTAATACCTATTATAATTGGAGGATTTGGAAATTGATTAGTTCCTCTAATATTAGGA   225
V2      ATAGTAATACCTATTATAATTGGAGGATTTGGAAATTGATTAGTTCCTCTAATATTAGGA   240
V3      ATAGTAATACCTATTATAATTGGAGGATTTGGAAATTGATTAGTTCCTCTAATATTAGGA   228
N5      ATAGTAATACCTATTATAATTGGAGGATTTGGAAATTGATTAGTTCCTCTAATATTAGGA   226
KC5     ATAGTAATACCTATTATAATTGGAGGATTTGGAAATTGATTAGTTCCTCTAATATTAGGA   231
        ************************************************************

P4      GCACCAGATATAGCCTTTCCTCGAATAAATAATATAAGTTTTTGAATATTACCTCCTTCA   288
P3      GCACCAGATATAGCCTTTCCTCGAATAAATAATATAAGTTTTTGAATATTACCTCCTTCA   288
KC1     GCACCAGATATAGCCTTTCCTCGAATAAATAATATAAGTTTTTGAATATTACCTCCTTCA   285
V2      GCACCAGATATAGCCTTTCCTCGAATAAATAATATAAGTTTTTGAATATTACCTCCTTCA   300
V3      GCACCAGATATAGCCTTTCCTCGAATAAATAATATAAGTTTTTGAATATTACCTCCTTCA   288
N5      GCACCAGATATAGCCTTTCCTCGAATAAATAATATAAGTTTTTGAATATTACCTCCTTCA   286
KC5     GCACCAGATATAGCCTTTCCTCGAATAAATAATATAAGTTTTTGAATATTACCTCCTTCA   291
        ************************************************************

P4      TTGACACTGCTACTTTCAAGTAGTATAGTAGAAAATGGATCAGGGACAGGATGAACAGTT   348
P3      TTGACACTGCTACTTTCAAGTAGTATAGTAGAAAATGGATCAGGGACAGGATGAACAGTT   348
KC1     TTGACACTGCTACTTTCAAGTAGTATAGTAGAAAATGGATCAGGGACAGGATGAACAGTT   345
V2      TTGACACTGCTACTTTCAAGTAGTATAGTAGAAAATGGATCAGGGACAGGATGAACAGTT   360
V3      TTGACACTGCTACTTTCAAGTAGTATAGTAGAAAATGGATCAGGGACAGGATGAACAGTT   348
N5      TTGACACTGCTACTTTCAAGTAGTATAGTAGAAAATGGATCAGGGACAGGATGAACAGTT   346
KC5     TTGACACTGCTACTTTCAAGTAGTATAGTAGAAAATGGATCAGGGACAGGATGAACAGTT   351
        ************************************************************

P4      TATCCACCTCTTTCATCTGGAACTGCCCATGCAGGAGCTTCAGTAGACTTAACAATTTTT   408
P3      TATCCACCTCTTTCATCTGGAACTGCCCATGCAGGAGCTTCAGTAGACTTAACAATTTTT   408
KC1     TATCCACCTCTTTCATCTGGAACTGCCCATGCAGGAGCTTCAGTAGACTTAACAATTTTT   405
V2      TATCCACCTCTTTCATCTGGAACTGCCCATGCAGGAGCTTCAGTAGACTTAACAATTTTT   420
V3      TATCCACCTCTTTCATCTGGAACTGCCCATGCAGGAGCTTCAGTAGACTTAACAATTTTT   408
N5      TATCCACCTCTTTCATCTGGAACTGCCCATGCAGGAGCTTCAGTAGACTTAACAATTTTT   406
KC5     TATCCACCTCTTTCATCTGGAACTGCACATGCAGGAGCTTCAGTAGACTTAACAATTTTT   411
```

```
         ************************** *********************************

P4       TCTCTTCATTTAGCAGGAGTATCATCAATTTTAGGAGCAGTAAATTTTATTACTACTGTT   468
P3       TCTCTTCATTTAGCAGGAGTATCATCAATTTTAGGAGCAGTAAATTTTATTACTACTGTT   468
KC1      TCTCTTCATTTAGCGGGAGTATCATCAATTTTAGGAGCAGTAAATTTTATTACTACTGTT   465
V2       TCTCTTCATTTAGCGGGAGTATCATCAATTTTAGGAGCAGTAAATTTTATTACTACTGTT   480
V3       TCTCTTCATTTAGCGGGAGTATCATCAATTTTAGGAGCAGTAAATTTTATTACTACTGTT   468
N5       TCTCTTCATTTAGCAGGAGTATCATCAATTTTAGGAGCAGTAAATTTTATTACTACTGTT   466
KC5      TCTCTTCATTTAGCAGGAGTATCATCAATTTTAGGAGCAGTAAATTTTATTACTACTGTT   471
         ************** *********************************************

P4       ATTAATATACGATCTGCAGGAATTACTTTAGATCGATTACCTTTATTTGTTTGATCTGTA   528
P3       ATTAATATACGATCTGCAGGAATTACTTTAGACCGATTACCTTTATTTGTTTGATCTGTA   528
KC1      ATTAATATACGATCTGCAGGAATTACTTTAGACCGATTACCTTTATTTGTTTGATCTGTA   525
V2       ATTAATATACGATCTGCAGGAATTACTTTAGACCGATTACCTTTATTTGTTTGATCTGTA   540
V3       ATTAATATACGATCTGCAGGAATTACTTTAGACCGATTACCTTTATTTGTTTGATCTGTA   528
N5       ATTAATATACGATCTGCAGGAATTACTTTAGACCGATTACCTTTATTTGTTTGATCTGTA   526
KC5      ATTAATATACGATCTGCAGGAATTACTTTAGACCGATTACCTTTATTTGTTTGATCTGTA   531
         ******************************** ***************************

P4       GTAATTACAGCTGTATTATTACTTTTATCATTACCTGTATTAGCCGGAGCTATTACTATA   588
P3       GTAATTACAGCTGTATTATTACTTTTATCATTACCTGTATTAGCCGGAGCTATTACTATA   588
KC1      GTAATTACAGCTGTATTATTACTTTTATCATTACCTGTATTAGCCGGAGCTATTACTATA   585
V2       GTAATTACAGCTGTATTATTACTTTTATCATTACCTGTATTAGCCGGAGCTATTACTATA   600
V3       GTAATTACAGCTGTATTATTACTTTTATCATTACCTGTATTAGCCGGAGCTATTACTATA   588
N5       GTAATTACAGCTGTATTATTACTTTTATCATTACCTGTATTAGCCGGAGCTATTACTATA   586
KC5      GTAATTACAGCTGTATTATTACTTTTATCATTACCTGTATTAGCCGGAGCTATTACTATA   591
         ************************************************************

P4       TTATTAACTGATCGAAATTTAAATACTTCATTCTTTGACCCAATTGGAGGAGGGGATCCT   648
P3       TTATTAACTGATCGAAATTTAAATACTTCATTCTTTGACCCAATTGGAGGAGGGGATCCT   648
KC1      TTATTAACTGATCGAAATTTAAATACTTCATTCTTTGACCCAATTGGAGGAGGGGATCCT   645
V2       TTATTAACTGATCGAAATTTAAATACTTCATTCTTTGACCCAATTGGAGGAGGGGATCCT   660
V3       TTATTAACTGATCGAAATTTAAATACTTCATTCTTTGACCCAATTGGAGGAGGGGATCCT   648
N5       TTATTAACTGATCGAAATTTAAATACTTCATTCTTTGACCCAATTGGAGGAGGTGACCCT   646
KC5      TTATTAACTGATCGAAATTTAAATACTTCATTCTTTGACCCAATTGGAGGAGGTGACCCT   651
         ***************************************************** ** ***

P4       ATTTTATATCAACATCTATTTTGAT-------------------   673
P3       ATTTTATATCAACATCTATTTTGATTTTTT--------------   678
KC1      ATTTTATATCAACATCTATTTTGA--------------------   669
V2       ATTTTATATCAACATCTATTTTGATTTTTTGGTCACCTGGA---   701
V3       ATTTTATATCAACATCTATTTTGATT------------------   674
N5       ATTTTATATCAACATCTATTTTGATTTTTTGG------------   678
KC5      ATTTTATATCAACATCTATTTTGATTTTTTGGTCACCCGGAAAA   695
         ************************
```
