# Supplementary material for: The monsoon-associated equine South African pointy mosquito ‘Aedes caballus’; the first comprehensive record from southeastern Iran with a description of ecological, morphological, and molecular aspects
Source: PLoS One. 2024 May 23;19(5):e0298412. doi: 10.1371/journal.pone.0298412 (PMC11115297; doi:10.1371/journal.pone.0298412)
Supplement: S2 Fig — ITS2 locus sequences with a diversity rate of 1.6%, characterized by six mutations, including two transitions and four transversions. (PDF) [file pone.0298412.s002.pdf]

```
CLUSTAL O(1.2.4) multiple sequence alignment


A06      CGTGGATCGATGAAGACCGCAGCTAAATGCGCGTCAGAATGTGAACTGCAGGACACATGA   60
A20      CGTGGATCGATGAAGACCGCAGCTAAATGCGCGTCAGAATGTGAACTGCAGGACACATGA   60
A23      CGTGGATCGATGAAGACCGCAGCTAAATGCGCGTCAGAATGTGAACTGCAGGACACATGA   60
A03      CGTGGATCGATGAAGACCGCAGCTAAATGCGCGTCAGAATGTGAACTGCAGGACACATGA   60
A25      CGTGGATCGATGAAGACCGCAGCTAAATGCGCGTCAGAATGTGAATTGCAGGACACATGA   60
         ********************************************* **************

A06      ACACCGACAAGTTGAACGCATATTGCACATCGTACAACAGTACGATGTACACATTTTTGA   120
A20      ACACCGACAAGTTGAACGCATATTGCACATCGTACAACAGTACGATGTACACATTTTTGA   120
A23      ACACCGACAAGTTGAACGCATATTGCACATCGTACAACAGTACGATGTACACATTTTTGA   120
A03      ACACCGACAAGTTGAACGCATATTGCACATCGTACAACAGTACGATGTACACATTTTTGA   120
A25      ACACCGACAAGTTGAACGCATATTGCACATCGTACAACAGTACGATGTACACATTTTTGA   120
         ************************************************************

A06      GTGCCTATATTTATCCATTCAACTATACGTGCGTGCGCGTTTCACTTCGGGTGGACAGGC   180
A20      GTGCCTATATTTATCCATTCAACTATACGTGCGTGCGCGTTTCACTTCGGGTGGACAGGC   180
A23      GTGCCTATATTTATCCATTCAACTATACGTGCGTGCGCGTTTCACTTCGGGTGGACAGGC   180
A03      GTGCCTATATTTATCCATTCAACTATACGTGCGTGCGCGTTTCACTTCGGGTGGACAGGC   180
A25      GTGCCTATATTTATCCATTCAACTATACGTGCGTGCGCGTTTCACTTCGGGTGGACAGGC   180
         ************************************************************

A06      GCACGGCCCATAAGCACGTATGCGTAGTGACGTTTTTCCGCCTTCAGTGGTGGCAAAACG   240
A20      GCACGGCCCATAAGCACGTATGCGTAGTGACGTTTTTCCGCCTTCAGTGGTGGCAAAACG   240
A23      GCACGGCCCATAAGCACGTATGCGTAGTGACGTTTTTCCGCCTTCGGTGGTGGCAAAACG   240
A03      GCACGGCCCATAAGCACGTATGCGTAGTGACGTTTTTCCGCCTTCGGTGGTGGCAAAACG   240
A25      GCACGGCCCATAAGCACGTATGCGTAGTGACGTTTTTCCGCCTTCGGTGGTGGCAAAACG   240
         ********************************************* **************

A06      TTTAAGATAGTCAGGCGCGTCCTCCCCGTCCTCGGGCGGGTGCGGACGTGGTTGATGAAT   300
A20      TTTAAGATAGTCAGGCGCGTCCTCCCCGTCCTCGGGCGGGTGCGGACGTGGTTGATGAAT   300
A23      TTTAAGATAGTCAGGCGCGTCCTCCCCGTCCTCGGGCGGGTGCGGACGTGGTTGATGAAT   300
A03      TTTAAGATAGTCAGGCGCGTCCTCCCCGTCCTCGGGCGGGTGCGGACGTGGTTGATGAAT   300
A25      TTTAAGATAGTCAGGCGCGTCCTCCCCGTCCTCGGGCGGGTGCGGACGTGGTTGATGAAT   300
         ************************************************************

A06      ACATCCCCTAGACCACACCATCGGTTGGTTATGTTGTATTTCATCGGCACCACCTATCCT   360
A20      ACATCCCCTAGACCACACCATCGGTTGGTTATGTTGTATTTCATCGGCACCACCTATCCT   360
A23      ACATCCCCTAGACCACACCATCGGTTGGTTATGTTGTATTTCATCGGCACCACCTATCCT   360
A03      ACATCCCCTAGACCACACCATCGGTTGGTTATGTTGTATTTCATCGGCACCACCTATCCT   360
A25      ACATCCCCTAGACCACACCATCGGTTGGTTATGTTGTATTTCATCGGCACCACCTATCCT   360
         ************************************************************

A06      ATCGA-------CATTACACCAGTAGGCCTCAAATAATGTGTGAC   398
A20      ATCGA-------CATTACACCAGTAGGCCTCAAATAATGTGTGAC   398
A23      CTATCGACATTACATTACACCAGTAGGCCTCAAATAATGTGTGAC   405
A03      CTATCGACATTACATTACCAGTA--GGCCTCAAATAATGTGTGAC   403
A25      CTATCGACATTACATTACCAGTA--GGCCTCAAATAATGTGTGAC   403
          *          ******       ********************
```
