# Supplementary material for: The monsoon-associated equine South African pointy mosquito ‘Aedes caballus’; the first comprehensive record from southeastern Iran with a description of ecological, morphological, and molecular aspects
Source: PLoS One. 2024 May 23;19(5):e0298412. doi: 10.1371/journal.pone.0298412 (PMC11115297; doi:10.1371/journal.pone.0298412)

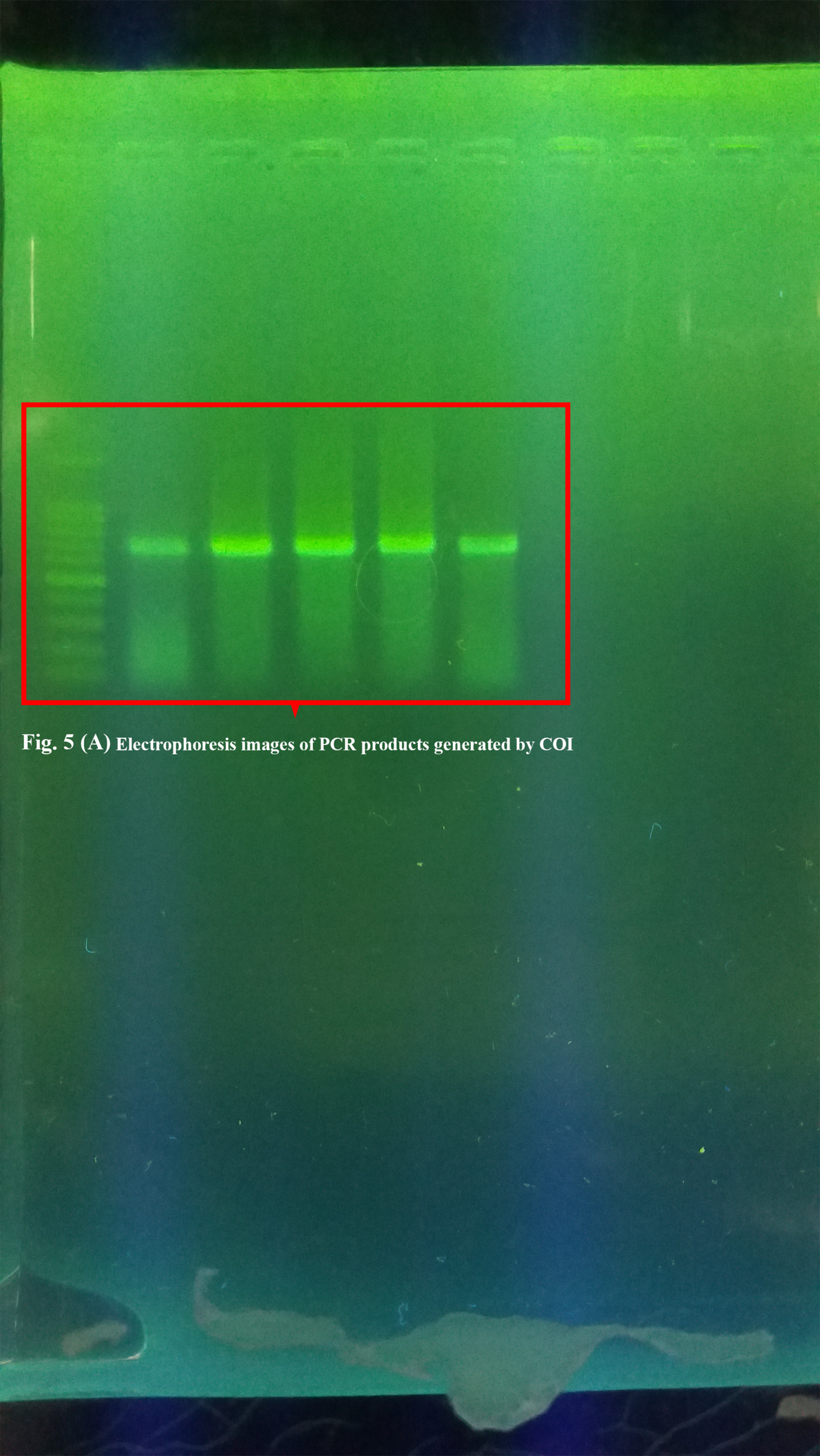

Fig. 5 (A) Electrophoresis images of PCR products generated by COI

Fig. 5 (B) Electrophoresis images of PCR products generated by ITS2

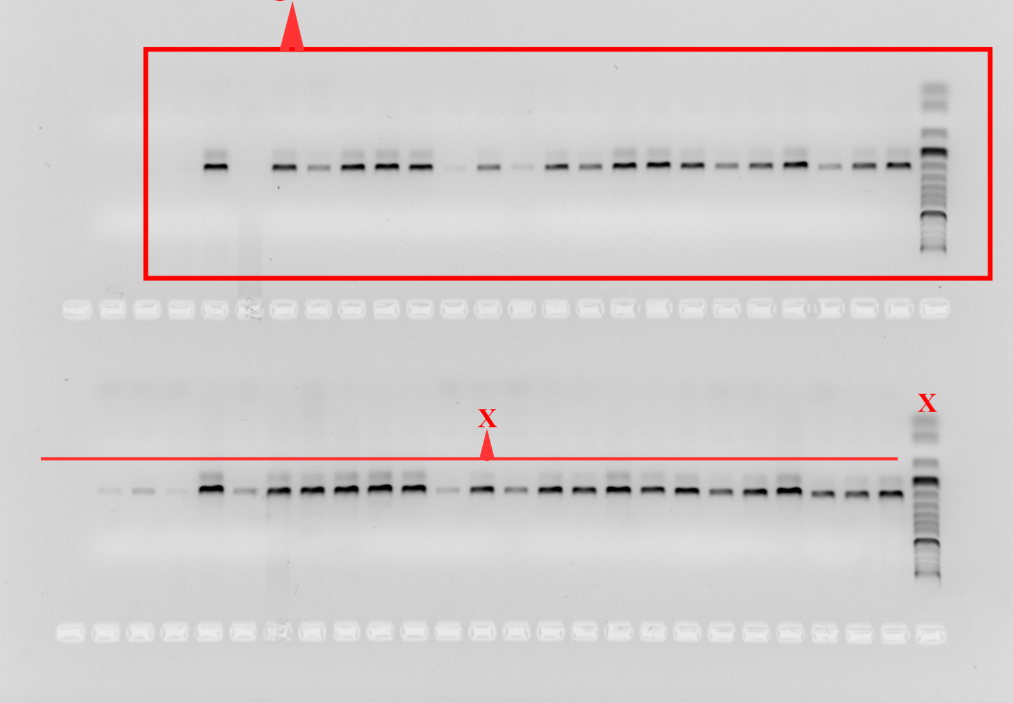

Fig. 5 (C) Electrophoresis images of PCR products generated by ANT

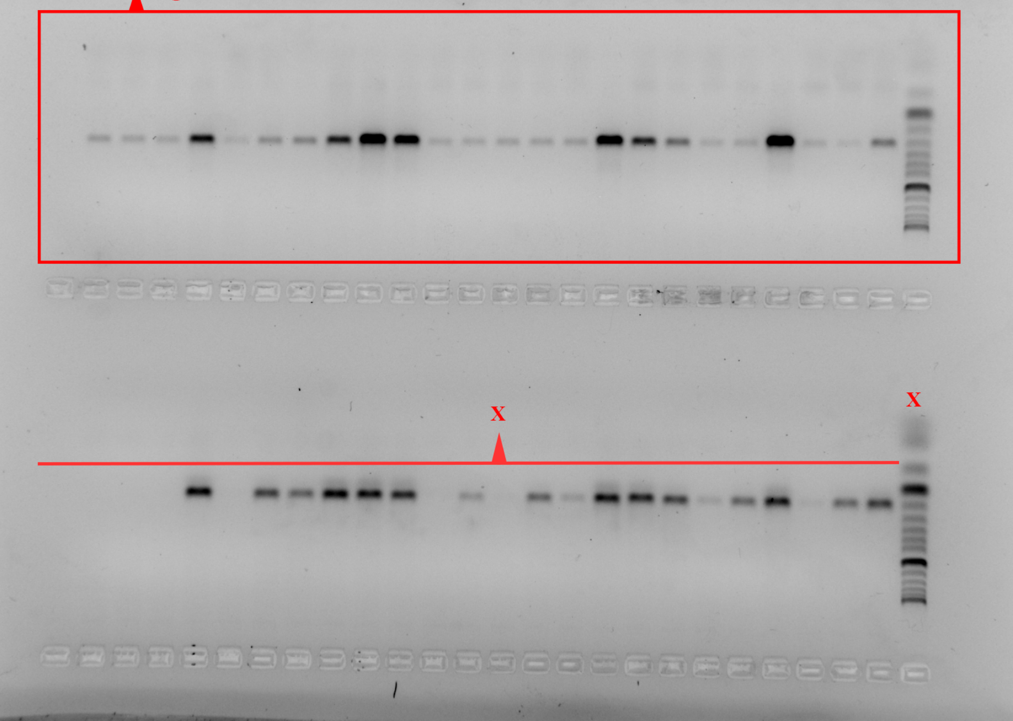

Supplement: S1 Raw images — (PDF) [file pone.0298412.s003.pdf]
